# Determinants of enduring major depressive episodes in the youth population of Hong Kong: The roles of comorbid psychopathology and stressful life events

**DOI:** 10.1017/S0033291725102468

**Published:** 2025-11-20

**Authors:** Stephanie Ming Yin Wong, Eric Yu Hai Chen, Yi Nam Suen, Jim van Os, Peter B. Jones, Patrick D. McGorry, Tai Hing Lam, Craig Morgan, David McDaid, Pak Chung Sham, Linda Chiu Wa Lam, Cindy Tsui, Charlton Cheung, Edwin Ho Ming Lee, Sherry Kit Wa Chan, Christy Lai Ming Hui

**Affiliations:** 1 Department of Social Work and Social Administration, The University of Hong Kong, Hong Kong SAR, PR China; 2 Centre for Youth Mental Health, Faculty of Medicine, Dentistry and Health Sciences, University of Melbourne, Parkville, VIC, Australia; 3 Orygen Youth Health, Parkville, VIC, Australia; 4 Department of Psychiatry, School of Clinical Medicine, LKS Faculty of Medicine, The University of Hong Kong, Hong Kong SAR, PR China; 5 School of Nursing, LKS Faculty of Medicine, The University of Hong Kong, Hong Kong SAR, PR China; 6 Department of Psychiatry, Utrecht University Medical Centre, Utrecht, The Netherlands; 7 Department of Psychiatry, University of Cambridge, Cambridge, UK; 8 School of Public Health, LKS Faculty of Medicine, The University of Hong Kong, Hong Kong SAR, PR China; 9 Health Service & Population Research Department, Institute of Psychiatry, Psychology, and Neuroscience, King’s College London, London, UK; 10 Care Policy and Evaluation Centre, Department of Health Policy, London School of Economics and Political Science, London, UK; 11 The State Key Laboratory of Brain and Cognitive Sciences, The University of Hong Kong, Hong Kong SAR, PR China; 12 Department of Psychiatry, Faculty of Medicine, The Chinese University of Hong Kong, Hong Kong SAR, PR China

**Keywords:** cohort study, generalized anxiety, major depressive episode, PTSD, risk factors, youth mental health

## Abstract

**Background:**

Major depressive episodes (MDEs) are highly recurrent in clinical samples. However, the course of MDEs and predictors of their endurance are unclear in the general youth population.

**Methods:**

We investigated prospective factors associated with enduring MDE (the presence of 12-month DSM-IV MDE at baseline and 1 year using the Composite International Diagnostic Interview–Screening Scales) in 1,833 participants of a 1-year epidemiological youth cohort study in Hong Kong. Multivariable logistic regression models were used to examine the influences of a range of personal and environmental factors.

**Results:**

At baseline, 13.7% participants had MDEs, among whom 21.1% presented enduring MDEs. More severe symptoms of post-traumatic stress disorder (adjusted odds ratio [aOR] = 5.54, confidence interval [CI] = 2.14–14.38), depression (aOR = 3.92, CI = 1.79–8.62), and generalized anxiety (aOR = 2.27, CI = 1.21–4.25) at baseline were among the strongest associated factors for enduring MDE, with trends of associations observed for psychotic-like experiences (aOR = 1.98, CI = 0.98–4.02) and eating disorder symptoms (aOR = 1.88, CI = 0.90–3.95). Among various types of stressors, only dependent stressors at follow-up showed a clear association with enduring MDE (aOR = 4.22, CI = 1.81–9.83). Those with enduring MDE showed poorer functioning and mental health-related quality of life at follow-up, with only 35.6% having sought any psychiatric/psychological help during the past year.

**Conclusions:**

Detecting comorbid symptoms in those with prior MDEs and reducing the impact of dependent stressors may help reduce their long-term implications. Enhancing the accessibility and acceptability of youth-targeted mental health services would also be crucial to improve help-seeking.

## Introduction

Depressive disorders are among the leading causes of disability-adjusted life years in young people (GBD 2019 Diseases and Injuries Collaborators, 2020). With the onset of most adult depressive disorders occurring before the age of 25 years (Solmi et al., 2022), early prevention and intervention initiatives targeting this age group have been called for (McGorry et al., 2024). Clinical studies have shown that around 50% of those with a history of depression may experience at least one recurrent episode in their lifetime (Benjet et al., 2020; Burcusa & Iacono, 2007). Yet, few studies have examined associated factors that could facilitate the early identification of those at risk of a more enduring course of depression in the general youth population.

External stressors and intrinsic vulnerabilities have been suggested to play differential roles in the course of depression, with the former being more predictive of its onset and the latter (such as depressive mood, depression-related memories, and neuroticism traits) more predictive of its chronicity (Beck, 1967; Monroe et al., 2019; Teasdale, 1988). Several other personal-level clinical and psychological factors, such as earlier onset, comorbid anxiety, and a lack of perceived social support, have also been conceived to be linked to a poorer course of depression (Burcusa & Iacono, 2007; Spijker et al., 2001). However, whether different types of external influences might differentially affect outcomes of depression has not been investigated.

Existing work has shown that people with prior experiences of a depressive episode tend to be more vulnerable to future stressful life events (SLEs), particularly those that arise partially due to one’s characteristics or behaviors, such as interpersonal conflicts and school dropout (Liu & Alloy, 2010; Technow et al., 2015). The cumulative experience of this form of self-generated life stressors, also known as dependent SLEs, can contribute to elevated risks of subsequent depressive episodes. The role of such dependent SLEs may be different from independent SLEs, which are those that occur outside of one’s control (e.g. natural disasters) (Hammen, 1991). Indeed, dependent SLEs have been reported to show stronger associations with depressive symptoms as compared with independent SLEs, partially due to the maladaptive self-inferences that tend to arise following the more personally relevant stressful experience in those with depressive and anxiety symptoms (Fassett-Carman et al., 2020). Nevertheless, studies on the chronicity of major depressive disorder in the general population have not differentiated between these types of SLEs (Sargeant et al., 1990; Spijker et al., 2001), the examination of which may provide a more nuanced understanding of the etiologies and possibly prevention of depressive episodes in young people.

To more accurately establish depression outcomes, the use of not only self-administered measures but also clinical diagnostic interviews – which allow for a more comprehensive assessment of clinical symptoms – is needed. To date, only one study conducted among young people has examined factors associated with the recurrence/persistence of depression using standardized diagnostic instruments (Benjet et al., 2020). This two-wave study (2005 and 2013) found a combination of personal (childhood onset of depression) and external (domestic violence) factors to be predictors of recurrent major depressive disorder, as defined according to the Diagnostic and Statistical Manual of Mental Disorders, fourth edition (DSM-IV), using the World Mental Health Composite International Diagnostic Interview (CIDI). Although a range of recent-onset traumatic events (TEs) and other psychiatric conditions were accounted for (e.g. anxiety disorder, substance use disorder, and eating disorder), the study did not capture other depression-related factors at baseline (e.g. symptom severity), which is among the most consistent predictors of poor depression outcomes previously identified (Buckman et al., 2018; Burcusa & Iacono, 2007).

More importantly, existing studies examining factors associated with depression outcomes using diagnostic instruments were conducted over 10 years ago. The rapid growth in the use of smartphones and social media apps, such as TikTok in 2018 and RedNote in 2019 (Hart, 2025; Paul, 2022), has been linked to the rise in mental health problems among young people (Hidaka, 2012; Twenge, 2020). Of note, their implications for health are further compounded by the increasing observations of overlapping global threats, such as political conflicts, pandemics and epidemics, climate change and natural hazards, and economic turmoil, also referred to as ‘polycrisis’ (Asbrand et al., 2023; Kwamie et al., 2024). The social and lifestyle changes experienced by this new generation of youths, as previously shown to have important mental health implications on populations (Amerio et al., 2020), need to be aptly addressed in an updated study.

As an effort to inform more timely detection and intervention of depression risks in the community, a household-based epidemiological study was conducted from 2019 to 2022 among young people aged 15–24 years in Hong Kong. A series of previous works has revealed the prevalence of past 12-month major depressive episode (MDE, 13.7%) and other major mental health and suicide-related outcomes, as well as the associations of psychological factors (e.g. lower resilience and loneliness), lifestyle factors (e.g. frequent nightmares and smartphone overuse), and stressors at both personal and population levels (e.g. personal SLEs, childhood adversity, exposure to social unrest and the coronavirus disease 2019 [COVID-19] pandemic) with clinically-relevant MDE (Hui et al., 2022; Suen et al., 2024; Wong, Chen et al., 2023; S. M. Y. Wong et al., 2023; Wong, Ip et al., 2022). The clinical outcomes of MDE in the youth population over time, as well as the factors that might contribute to the endurance of MDE, remain to be examined.

Building on these previous works, the present study first aimed to examine the rate of *enduring MDE* in this population-based youth cohort in Hong Kong, which we defined as the presence of DSM-based MDE both at baseline and at 1-year follow-up. Our second aim was to identify potential predictors of enduring MDE with consideration of a wide range of personal-level and external factors relevant to the societal changes undergone during the past decade.

We hypothesized that personal-level factors, such as more severe depressive symptoms, comorbid mental health problems, and neuroticism personality traits, would show the strongest associations with enduring MDE. Several modifiable psychological and lifestyle factors shown to be associated with depression outcomes were also examined for their links to enduring MDE in this study, including loneliness, hopelessness, impulsivity, and resilience, as well as family functioning, sleep, physical activity, and problematic smartphone use. Regarding the various external stressors, we anticipated that their associations with enduring MDE would vary according to the personal relevance of the events, with childhood adversity and recent dependent SLEs exerting the greatest effects, followed by independent SLEs and population-level events, such as social unrest-related TEs and COVID-19 pandemic-related events (PEs), which the Hong Kong population had been exposed to at the time of baseline data collection.

## Methods

### Study design and population

Data analyzed in this study were from the 1-year follow-up cohort of the Hong Kong Youth Epidemiological Study of Mental Health, which was a territory-wide, household-based study of youth mental health in the region. A stratified cluster sampling design was used: a random selection of addresses expected to be occupied by a young person aged 15–24 years (according to the latest By-census 2016) was obtained from the Census and Statistics Department of the Hong Kong SAR Government and stratified by geographic district and housing type. Invitation letters were then sent to these addresses, providing information about the study with the research team contact for participant recruitment. All local residents within this age range, who were able to provide informed consent, were eligible to ensure inclusivity. More details can be found in other published studies (Hui et al., 2022; Wong, Hui et al., 2023; C. S. M. Wong et al., 2023; Wong, Ip et al., 2022).

All data at baseline were collected from May 2019 to July 2022. Participants were invited to the follow-up study from late October 2020 to August 2023. Of the 2,110 participants reached, 1,833 (86.9% response) consented and participated in the follow-up interview (median follow-up duration = 13 months, interquartile range = 12–14 months). For both baseline and follow-up interviews, standardized assessments were conducted by trained research assistants through face-to-face interviews, with an option of online video conferencing during COVID-19, following the same procedures.

### Measures

#### Major depressive episodes

Enduring MDE was defined as the presence of an MDE during the previous 12 months, both at baseline and follow-up, similar to previous work (Wang et al., 2012). MDEs during the previous 12 months were assessed using the structured, interviewer-rated WHO CIDI–Screening Scales (CIDI-SC) (Kessler et al., 2013) based on the DSM-IV criteria at baseline and follow-up. The CIDI-SC is intended for population-based studies and has been adopted in our prior work (Wong, Chen et al., 2023), as well as the WHO World Mental Health Surveys International College Student Initiative (Auerbach et al., 2018). The age at MDE onset was also assessed using the CIDI-SC.

#### Risk and protective factors

Related personality and psychological factors at baseline were assessed, including neuroticism (neuroticism subscale of the Big Five Inventory; John et al., 2012; So et al., 2023), loneliness (UCLA Loneliness Scale, version 3; Ip et al., 2024; Russell, 1996), hopelessness (Beck Hopelessness Scale; Beck et al., 1974; Wong, Ip et al., 2022), impulsivity (Barratt Impulsiveness Scale version 11; Patton et al., 1995; Yao et al., 2007), and resilience (10-item Connor–Davidson Resilience Scale; Campbell-Sills & Stein, 2007; Rui et al., 2020).

Seven symptom dimensions were also assessed using validated measures, with more severe symptoms operationalized according to conventional cutoffs. They included symptoms of depression (Patient Health Questionnaire–9-item; Kroenke et al., 2001); generalized anxiety (Generalized Anxiety Disorder–7-item; Spitzer et al., 2006); psychotic-like experiences (PLEs) (Community Assessment of Psychic Experiences–Positive Scale; Capra et al., 2013); post-traumatic stress disorder (PTSD) (Trauma Screening Questionnaire; Brewin et al., 2002); alcohol dependence (Alcohol Use Disorders Identification Test; Conigrave et al., 1995); eating disorder (Eating Disorder Examination Questionnaire; Fairburn & Beglin, 1994); and social anxiety (Liebowitz Social Anxiety Scale; Heimberg et al., 1999).

Family functioning was assessed using the Brief Family Relationship Scale, in which a higher score reflects poorer family functioning (Fok et al., 2014). Poor sleep quality was assessed using the Pittsburgh Sleep Quality Index (PSQI) (Tsai et al., 2005). Frequent nightmares were operationalized as having ≥1 nightmare per week during the past month using an item from the PSQI (Li et al., 2010; Wong, Hui et al., 2023). Physical activity was operationalized as the number of days during the past week engaged in moderate-intensity and vigorous-intensity physical activity using two items from the International Physical Activity Questionnaire (Macfarlane et al., 2011; Wong, Chen et al., 2023). Problematic smartphone use was assessed using the modified version of the Revised Chen Internet Addiction Scale (Mak et al., 2014; Wong, Chen et al., 2022; Wong, Lau et al., 2023).

Childhood adversity before the age of 17 years was assessed using the CIDI 3.0 (Kessler & Ustün, 2004), which captures experiences related to emotional abuse, physical abuse, neglect, and sexual abuse through a structured interview. Responses to each item were rated on a 5-point scale (‘*never*’ to ‘*very often*’). A rating of 3 or above for any of these items (‘*sometimes*’ to ‘*very often*’) reflected prior childhood adversity exposure, as in previous work (Wong, Chen et al., 2023).

Exposure to population-level environmental stressors in recent years in Hong Kong was assessed using a series of locally adapted items (Wong, Chen et al., 2023). Significant social unrest events since June 2019 included ‘crowd dispersal by the use of force’, ‘arrest or detention’, and ‘media viewing of others being physically attacked’. Significant experiences related to the pandemic since its outbreak in January 2020 included ‘having sufficient gears’ (reversed), ‘increased personal and rest time due to remote work/school’ (reversed), ‘increased conflicts with family due to remote work/school’, and ‘increased work/studies hours due to remote work/school’ (see Supplementary Table S1 for details).

All the above risk and protective factors were assessed at baseline. In addition, exposure to recent personal SLEs during the past 12 months was assessed at follow-up using the List of Threatening Events adapted to the local youth population (Brugha et al., 1985; Wong, Chen et al., 2023). For instance, ‘expelled from school’ and ‘dropped out of school’ were provided as alternatives to ‘sacked from job’ and ‘unemployment’, respectively. Seven of these SLEs were categorized as dependent SLEs (e.g. ‘serious problem with a close friend, neighbor, or relative’ and ‘major financial crisis’), while five of them were categorized as independent SLEs (e.g. ‘serious illness, injury, or assault to a close relative’, and ‘death of first-degree relative’ (Brugha et al., 1985).

#### Functioning

Functional impairment due to psychiatric symptoms at follow-up was assessed using two items that capture the number of days with reduced and lost productivity during the past 30 days (S. M. Y. Wong, Chen et al., 2023). The interviewer-rated Social and Occupational Functioning Assessment Scale (Goldman et al., 1992) was used to assess the degree of social and occupational functioning during the past 6 months. Physical and mental health-related quality of life (HR-QoL) were assessed using the physical and mental components of the 12-Item Short Form Health Survey, respectively (Ware et al., 1995). Service utilization was defined as receiving any services from a psychiatrist, psychologist, or community psychiatric nurse during the past 12 months (Wong, Chen et al., 2023).

All measures of the study have been adopted in the Chinese youth populations, with more details of their psychometric properties provided in Supplementary Table S1. Sociodemographic information, including biological sex (male/female), age, socioeconomic status (reflected by any government subsidy received) (Wong, Chen et al., 2023), and any family psychiatric history, was also captured.

### Statistical analysis

Participant characteristics in the complete-case sample, including the rate of enduring MDE, were first described. The distribution of the various domains of risk and protective factors was compared between those who showed enduring and remitted MDEs at follow-up using Chi-squared tests and Mann–Whitney *U*-tests for categorical and continuous variables, respectively. Using univariate and multivariable logistic regression models, we explored associations of the range of personality and psychological, clinical, family, and lifestyle factors, as well as external stressors, with enduring MDE, adjusting for the effects of sex, age, socioeconomic status, and family psychiatric history. Missing data were observed only in a small proportion of the sample across the variables tested (0%–4.4%; Supplementary Table S2). Nevertheless, to optimize the use of information collected from participants, we performed multiple imputation by chained equations with 20 imputations (using the *R* package *mice*) (Azur et al., 2011; Weavers et al., 2021), with estimates pooled following Rubin’s rule (Little & Rubin, 2019). The main study findings were based on analyses conducted on the imputed sample. To confirm the robustness of the findings, sensitivity analyses were conducted on the complete-case sample. Effect sizes, reflecting the strengths of associations, were presented in the form of unadjusted or adjusted odds ratios (OR and aOR) with 95% confidence intervals (CIs). All continuous variables in the logistic regression models were standardized to *Z*-scores to facilitate interpretation of the findings (Mars et al., 2019). Lastly, differences in functioning, HR-QoL, and rates of service utilization at follow-up were compared between those with enduring and remitted MDEs using Chi-squared tests and Mann–Whitney *U*-tests, respectively. All analyses were conducted using *R* (version 4.4.1).

### Ethical statement

Ethics approval for the study was granted by the Institutional Review Board of The University of Hong Kong/Hospital Authority Hong Kong West Cluster. Written informed consent was obtained from all participants, with parental or guardian consent additionally obtained from those below the age of 18 years. All procedures complied with the Helsinki Declaration of 1975 (revised in 2008). The STROBE reporting guideline was followed.

## Results

Of 1,833 young people who consented and participated in the follow-up interview (86.9% response), 1,620 (88.4%) had complete data on the variables of interest at 1-year follow-up. Sociodemographic variables, as well as the rate of enduring MDE, were comparable between participants with and without missing data (Supplementary Table S3). The complete-case sample comprised 58.2% (*n* = 943) females and had a mean age of 19.8 years (standard deviation = 2.7) at baseline (Table 1).

**Table 1.** Characteristics of the youth cohort, without and with enduring MDE at follow-up in the complete-case sample

	Sample with MDE at baseline (*n* = 222)	MDE outcomes at follow-up	
		Remitted^a^ (*n* = 177)	Enduring^b^ (*n* = 45)
**Sociodemographics**			
Female sex	155 (69.8%)	123 (69.5%)	32 (71.1%)
Age, mean (SD)	20.22 (2.71)	20.1 (2.7)	20.5 (2.6)
Any government subsidy received	19 (8.6%)	14 (7.9%)	5 (11.1%)
Any family psychiatric history	49 (22.1%)	39 (22.0%)	10 (22.2%)
**Personality and psychological factors**			
Neuroticism (BFI–Neuroticism), mean (SD)	30.45 (4.71)	30.11 (4.62)	31.78 (4.90)
Loneliness (UCLA-LS), mean (SD)	49.31 (9.27)	48.75 (9.38)	51.49 (8.60)
Hopelessness (BHS), mean (SD)	9.32 (4.31)	9.02 (4.20)	10.53 (4.55)
Impulsivity (BIS–11), mean (SD)	65.91 (8.55)	65.68 (8.46)	66.80 (8.93)
Resilience (CD-RISC–10), mean (SD)	20.11 (6.78)	20.47 (6.64)	18.69 (7.17)
**Clinical factors**			
Age at first MDE onset (CIDI SC), mean (SD)	15.89 (4.11)	16.0 (4.3)	15.8 (3.2)
Depressive symptoms (PHQ–9 ≥ 10)	133 (59.9%)	95 (53.7%)	38 (84.4%)
Generalized anxiety symptoms (GAD–7 ≥ 10)	89 (40.1%)	63 (35.6%)	26 (57.8%)
PLEs (CAPE-P15 frequency ≥ 1.57 and distress ≥1.17)	45 (20.3%)	32 (18.1%)	13 (28.9%)
PTSD symptoms (TSQ ≥ 6)	18 (8.1%)	9 (5.1%)	9 (20.0%)
Alcohol dependence symptoms (AUDIT ≥8)	21 (9.5%)	15 (8.5%)	6 (13.3%)
Eating disorder symptoms (EDE-Q mean ≥ 2.3)	42 (18.9%)	28 (15.8%)	14 (31.1%)
Social anxiety symptoms (LSAS ≥60)	73 (32.9%)	57 (32.2%)	16 (35.6%)
**Family and lifestyle factors**			
Poor family functioning (BFRS), mean (SD)	22.77 (8.23)	22.33 (8.35)	24.47 (7.60)
Poor sleep quality (PSQI), mean (SD)	7.64 (3.65)	7.69 (3.70)	7.40 (3.47)
Frequent nightmares (≥1/week)	76 (34.2%)	57 (32.2%)	19 (42.2%)
Days of physical activity (IPAQ), mean (SD)	2.77 (2.36)	2.86 (2.39)	2.42 (2.19)
Problematic smartphone use (CIAS-R ≥ 67)	107 (48.2%)	87 (49.2%)	20 (44.4%)
**External stressors**			
Any past exposure to childhood adversity	125 (56.3%)	98 (55.4%)	27 (60%)
≥2 social unrest-related TEs at baseline	56 (25.2%)	42 (23.7%)	14 (31.1%)
≥2 COVID–19 PEs at baseline^c^	90 (44.3%)	72 (43.9%)	18 (46.2%)
Recent stress exposure at follow-up (≥2 SLEs)	56 (25.2%)	37 (20.9%)	19 (42.2%)
≥2 independent SLEs at follow-up	25 (11.3%)	19 (10.7%)	6 (13.3%)
≥2 dependent SLEs at follow-up	23 (10.4%)	12 (6.8%)	11 (24.4%)

*Note.* Data are presented as *n* (%) or mean (SD).

Abbreviations: AUDIT, Alcohol Use Disorders Identification Test; BFI, Big Five Inventory; BFRS, Brief Family Relationship Scale; BHS, Beck Hopelessness Scale; BIS-11, Barratt Impulsiveness Scale-11; CAPE-P15, 15-item Community Assessment of Psychic Experiences-Positive Scale; CD-RISC-10, Connor–Davidson Resilience Scale 10-item; CIAS-R, Revised Chen Internet Addiction Scale; EDE-Q, Eating Disorder Examination Questionnaire; GAD-7, Generalized Anxiety Disorder scale; IPAQ, International Physical Activity Questionnaire; LSAS, Liebowitz Social Anxiety Scale; MDE, major depressive episode; PEs, COVID-19 pandemic-related events; PHQ-9, Patient Health Questionnaire; PSQI, Pittsburgh Sleep Quality Index; SLEs, personal stressful life events; TEs, social unrest-related traumatic events; TSQ, Trauma Screening Questionnaire; UCLA-LS, UCLA Loneliness Scale (Version 3).

a Absence of MDE at 1-year follow-up, assessed using the CIDI-SC, among those with 12-month MDE at baseline.

b Presence of MDE at 1-year follow-up, among those with 12-month MDE at baseline.

c Missing from 19 participants.

At baseline, 13.7% (*n* = 222) met the criteria for MDE during the previous 12 months. Compared to those without MDE at baseline, this group of young people presented significantly higher levels of risk across the various personality, psychological, clinical, family and lifestyle, and external stress domains at baseline (see Supplementary Table S4 for details). Among them, 20.3% (*n* = 45) showed enduring MDE at 1-year follow-up.

### Prospective factors associated with enduring MDE

Table 2 shows associations between the risk and protective factors and enduring MDE at follow-up. In both univariate and multivariable models, the strongest evidence of an association with enduring MDE among baseline measures was more severe PTSD symptoms (aOR = 5.54, CI = 2.14–14.38), followed by symptoms of depression (aOR = 3.92, CI = 1.79–8.62) and generalized anxiety (aOR = 2.27, CI = 1.21–4.25). Further, PLEs (aOR = 2.01, CI = 0.99–4.06) and eating disorder symptoms (aOR = 1.94, CI = 0.94–4.01) at baseline also showed trends of significant associations with enduring MDE. Aside from baseline factors, additional exposure to SLEs during the follow-up period was also associated with enduring MDE, with dependent SLEs showing stronger effects (aOR = 4.22, CI = 1.81–9.83) as compared with independent SLEs (aOR = 2.08, CI = 0.90–4.83). None of the distal stressors showed clear associations with enduring MDE at follow-up (Table 2). The effect sizes observed were comparable in the complete-case analysis (Supplementary Table S5).

**Table 2.** Risk and protective factors for enduring MDE

	Enduring MDE at follow-up				
	Unadjusted OR (95% CI)	*p*	Adjusted OR (95% CI)	*p*
**Sociodemographics**				
Female sex	1.08 (0.56–2.09)	0.811	1.06 (0.55–2.05)	0.862
Age	1.09 (0.80–1.48)	0.576	1.10 (0.81–1.50)	0.535
Any government subsidy received	1.27 (0.48–3.36)	0.635	1.32 (0.49–3.59)	0.583
Any family psychiatric history	1.14 (0.56–2.33)	0.710	1.14 (0.56–2.33)	0.719
**Personality and psychological factors**				
Neuroticism (BFI–Neuroticism)	1.27 (0.92–1.76)	0.145	1.28 (0.91–1.82)	0.160
Loneliness (UCLA-LS)	1.21 (0.91–1.60)	0.192	1.22 (0.91–1.63)	0.175
Hopelessness (BHS)	1.24 (0.95–1.62)	0.106	1.26 (0.95–1.67)	0.102
Impulsivity (BIS–11)	1.03 (0.79–1.34)	0.836	1.03 (0.78–1.36)	0.811
Resilience (CD-RISC–10)	0.86 (0.67–1.11)	0.258	0.86 (0.66–1.13)	0.277
**Clinical factors**				
Age at first MDE onset (CIDI-SC)	0.92 (0.07–1.20)	0.528	0.87 (0.65–1.18)	0.374
Depressive symptoms (PHQ–9 ≥ 10)	**3.89 (1.78–8.48)**	**0.001**	**3.92 (1.79–8.62)**	**0.001**
Generalized anxiety symptoms (GAD–7 ≥ 10)	**2.29 (1.23–4.27)**	**0.009**	**2.27 (1.21–4.25)**	**0.010**
PLEs (CAPE-P15 frequency ≥ 1.57 and distress ≥1.17)	1.94 (0.97–3.86)	0.060	2.01 (0.99–4.06)	0.052
PTSD symptoms (TSQ ≥ 6)	**5.48 (2.13–14.1)**	**<.001**	**5.54 (2.14–14.38)**	**<.001**
Alcohol dependence symptoms (AUDIT ≥8)	1.46 (0.54–3.96)	0.451	1.46 (0.53–4.01)	0.463
Eating disorder symptoms (EDE-Q mean ≥ 2.3)	1.96 (0.96–3.97)	0.063	1.94 (0.94–4.01)	0.073
Social anxiety symptoms (LSAS ≥60)	1.23 (0.65–2.34)	0.517	1.20 (0.62–2.30)	0.590
**Family and lifestyle factors**				
Poor family functioning (BFRS)	1.18 (0.92–1.53)	0.193	1.18 (0.91–1.53)	0.215
Poor sleep quality (PSQI)	1.02 (0.80–1.31)	0.871	1.01 (0.78–1.31)	0.914
Frequent nightmares (≥1/week)	1.57 (0.85–2.91)	0.150	1.59 (0.85–2.97)	0.146
Days of physical activity (IPAQ)	0.88 (0.64–1.21)	0.437	0.88 (0.63–1.23)	0.454
Problematic smartphone use (CIAS-R ≥ 67)	1.05 (0.57–1.93)	0.874	1.06 (0.57–1.96)	0.857
**Distal and recent stressors**				
Any past exposure to childhood adversity	1.34 (0.72–2.49)	0.357	1.30 (0.68–2.47)	0.432
≥2 social unrest-related TEs at baseline	1.34 (0.68–2.65)	0.400	1.33 (0.66–2.71)	0.425
≥2 COVID–19 PEs at baseline	0.97 (0.50–1.87)	0.922	0.97 (0.50–1.89)	0.935
Recent stress exposure at follow-up (≥2 SLEs)	**2.95 (1.55–5.62)**	**0.001**	**3.13 (1.61–6.05)**	**0.001**
≥2 independent SLEs at follow-up	2.03 (0.88–4.66)	0.094	2.08 (0.90–4.83)	0.087
≥2 dependent SLEs at follow-up	**4.15 (1.80–9.53)**	**0.001**	**4.22 (1.81–9.83)**	**0.001**

*Note.* Analyses were conducted on the imputed sample (*n* = 1,833). Sex, age, socioeconomic status (any government subsidy received), and family psychiatric history were adjusted for in the multivariable model. Statistics significant at the p < 0.05 level are in boldface.

Abbreviations: AUDIT, Alcohol Use Disorders Identification Test; BFI, Big Five Inventory; BFRS, Brief Family Relationship Scale; BHS, Beck Hopelessness Scale; BIS-11, Barratt Impulsiveness Scale-11; CAPE-P15, 15-item Community Assessment of Psychic Experiences-Positive Scale; CD-RISC-10, Connor-Davidson Resilience Scale 10-item; CIAS-R, Revised Chen Internet Addiction Scale; EDE-Q, Eating Disorder Examination Questionnaire; GAD-7, Generalized Anxiety Disorder scale; IPAQ, International Physical Activity Questionnaire; LSAS, Liebowitz Social Anxiety Scale; MDE, major depressive episode; PEs, COVID-19 pandemic-related events; PHQ-9, Patient Health Questionnaire; PSQI, Pittsburgh Sleep Quality Index; SLEs, personal stressful life events; TEs, social unrest-related traumatic events; TSQ, Trauma Screening Questionnaire; UCLA-LS, UCLA Loneliness Scale (Version 3).

### Functioning, health-related quality of life, and enduring MDE

In comparison with young people who showed remitted MDE, those who experienced an enduring course had more days of reduced (mean = 9.33 [SD] = 9.78) vs. 4.08 [6.09]) and lost (3.68 [7.15] vs. 0.63 [2.50]) productivity, as well as poorer social and occupational functioning (65.77 [11.24] vs. 75.38 [9.25]), all *p* < 0.001 (Supplementary Table S6). They also showed poorer mental health-related QoL (mean = 33.89 [SD = 13.62] vs. 43.10 [9.72]), with no significant difference observed in their physical health-related QoL.

### Past-year service utilization among young people with enduring MDE

Among those with MDE at baseline in the complete-case sample (*n* = 222), only 14.8% (*n* = 33) sought any psychiatric or psychological services during the past year, with a higher rate observed among those with enduring MDE (35.6%, *n* = 16) as compared with those who remitted (9.6%, *n* = 17), *p* < 0.001.

## Discussion

Using data from a prospective population-based youth sample, we explored factors associated with the endurance of MDE. Accounting for a comprehensive range of personal-level and external factors, we found evidence of not only more severe depressive symptoms at baseline to be prospectively associated with enduring MDE, but also comorbid symptoms of PTSD, generalized anxiety, PLEs, and eating disorders, as well as recent exposure to dependent SLEs. The presence of these factors in a young person with MDE should raise increased awareness of the need for more consistent treatment and monitoring to improve their prognosis.

The observation that around one in five young people have a more enduring course of MDE in our sample is slightly lower than those previously reported in the adult population using a similar study design (i.e. 1-year follow-up using clinical diagnostic measures for depression), such as the Epidemiologic Catchment Area (ECA) Study in the United States (23.5% after excluding attrition) (Sargeant et al., 1990), the NEMESIS in the Netherlands (28.3%) (Spijker et al., 2001), and a population-based study on working adults in Alberta, Canada (38.5%) (Wang et al., 2012). With the youth period being characterized as a ‘turbulent’ period of neurobiological development and psychosocial changes (Arain et al., 2013; Shorey et al., 2022), the lower but still significant level of chronicity of depressive symptomatology highlights the relevance of hope and optimism for recovery. This also reinforces the importance of early detection and treatment to avoid entrapment in a chronic depressive illness trajectory.

Consistent with these studies (Sargeant et al., 1990; Spijker et al., 2001; Wang et al., 2012), we found more severe depressive and generalized anxiety symptoms at baseline to be among the strongest predictors of enduring MDE. The observation that its endurance was most strongly associated with initial PTSD symptoms highlights the importance of accounting for prior trauma-related experiences and their impact on mental health. Aside from the existence of several shared risk factors (e.g. childhood adversity, neuroticism traits, and rumination) (Spinhoven et al., 2014; Wong et al., 2021b), the presence of PTSD symptoms in those with MDE might indicate alterations in attentional and memory processes (e.g. bias towards threat-related cues), which might have played a role in the prolonging of depressive symptoms. Our findings highlight the importance of considering the effects of prior trauma-related experiences on youth mental health. Of note, the presence of trauma and stress-related symptoms in a proportion of our youth sample might have reflected psychological responses to the series of co-occurring social unrest and COVID-19 pandemic-related stressors in Hong Kong at the time (Ni et al., 2020; Wong et al., 2021a). For others, these symptoms might also be indicative of pre-existing symptoms of PTSD or an elevated risk of the psychiatric disorder. Nevertheless, it should be emphasized that symptoms of PTSD were self-reported in this study and should not be conflated with the clinical disorder. How the presence of clinical PTSD, as assessed using diagnostic interviews, as well as its various symptom profiles, may contribute to enduring MDE should be explored in greater depth in future work.

Furthermore, this was among the first population-based studies to have examined the roles of PLEs and eating disorder symptomatology on the course of depression among young people. The observation that both of these symptom dimensions showed associations with enduring MDE even when adjusting for individual sociodemographic and family psychiatric history highlights the potential predictive value of the presence of these symptoms in MDE, suggesting the clinical relevance of including them as part of routine assessments of major depression. Indeed, the potential role of PLEs in signaling a more severe mood condition has been proposed (Chan et al., 2021; Perez & Jones, 2021). We do acknowledge that the various forms of psychotic symptoms and eating disorders could not be differentiated in the present study, which can be important given their differential biological underpinnings and prognosis. Examining how they may respectively contribute to the endurance of MDE, as well as determining whether these symptoms were primary or secondary to the depressive episode, can be crucial to inform more targeted intervention and treatment plans. Aside from extending a more rigorous diagnostic interview procedure for different disorders, adopting a symptom network approach may also be worthwhile to elucidate the interrelationships among individual symptoms across conventional diagnostic categories from a complex systems perspective (Borsboom, 2017).

Similar to findings in our previous work in the epidemiological youth sample (e.g. Hui et al., 2022; Suen et al., 2024; Wong, Chen et al., 2023; Wong, Hui et al., 2023; Wong, Ip et al., 2022), we did find significant cross-sectional associations between neuroticism trait, as well as the various psychological, family, and lifestyle factors, and 12-month MDE at baseline. However, contrary to our expectations, none of these factors was found to be associated with enduring MDE at follow-up. This observation might suggest that, while these factors might be indicative of the presence of a current depressive episode, intrinsic factors (as reflected by comorbid psychopathological symptoms in our study) might play a more vital role in determining its chronicity. It is also possible that these factors could have contributed to subclinical levels of depressive symptoms, which were not the focus of the present study but have been demonstrated in previous work (Kriesche et al., 2023; Pearce et al., 2022; Pedrelli et al., 2016; Wong, Chen et al., 2022). Future studies may build on our work to explore how the range of factors we examined may be associated with subclinical symptoms and the degree to which they may contribute to full-blown MDE. It would also be interesting to examine whether a similar set of factors would contribute to the new onset of MDE.

The finding that dependent, but not independent SLEs, showed significant influences on the endurance of MDE also provided an additional perspective on understanding the degree to which depression might be ‘maintained’ by external stressors. Cognitive models of enduring depression have suggested that, whereas external factors might trigger the onset of depression, intrinsic vulnerabilities play a stronger role in its maintenance (Lewinsohn et al., 1999; Monroe et al., 2019; Teasdale, 1988). Importantly, unlike independent SLEs, effective and timely treatment of MDE may reduce these self-generated stressors and potentially prevent the endurance of MDE. Of note is the potential relevance of population-level stressors for young people, which has become increasingly challenging in recent years, given the growing complexity of politico-economic upheavals globally (Hoyer et al., 2023; Kwamie et al., 2024). While we did not find their direct associations with the endurance of MDE in this study, a recent qualitative study has observed a substantial sense of powerlessness, heightened self-suppression, and feelings of uncertainty following such population-level threats among young people (To, 2025). Accounting for individual cognitive and psychosocial resources, as well as these broader determinants of health, would be needed to inform more person-centered care in future work.

Importantly, while we found substantially poorer functioning and mental HR-QoL at follow-up in those with enduring MDE, only one-third of these young people sought psychiatric or psychological help for their mental health needs. To reduce the burden associated with a chronic course of depression among the affected youths, their families, and the healthcare system, it is imperative to invest in research that examines barriers to service utilization and involve young people as part of the service design process.

## Strengths and limitations

To our best knowledge, this study was the first to investigate predictors of enduring MDE in a prospective population-based youth sample in the recent decade. A major strength is the consideration of a remarkably wide range of risk and protective factors, including psychopathological symptoms and various types of stressful events. Adding to early theoretical models of enduring depression, the exploration of how different factors may be similarly or differentially associated with MDE outcomes provided new information to further the understanding of the natural evolution of depression. This was also among the few studies that have investigated the real-world functional and HR-QoL consequences, as well as rates of service utilization, among young people with enduring MDE.

We also note several limitations. While the standardized CIDI-SC was used for assessing MDE, we cannot ascertain whether the outcome was a case of relapse, recurrence, or persistence. Similar to the ECA (Sargeant et al., 1990), the NEMESIS (Spijker et al., 2001), and the Alberta working adult population studies (Wang et al., 2012), we focused on examining the 1-year outcomes of MDE. We acknowledge that this follow-up period may be insufficient to capture the full clinical picture of depression and its evolution in young people. Indeed, early operational criteria have defined *relapse* as the occurrence of an MDE during a period of remission yet before recovery (2–8 weeks) and *remission* as the occurrence of a new episode after recovery (≥8 weeks asymptomatic) (Frank et al., 1991). Meanwhile, *persistent* depressive disorder is defined in the DSM-5-TR as the presence of depressive symptoms for at least 2 years, during which symptoms have not been absent for over 2 months (American Psychiatric Association, 2022). Since we were unable to distinguish between these outcomes, we used the term ‘enduring MDE’ to capture the presence of past 12-month MDE at both assessment time points. It would be important to further examine whether the risk and protective factors we identified would differ across these depression outcomes in a future study.

Further, with more data points available, additional statistical methods (e.g. latent class growth analysis) may also be used as supplementary to our present findings to \characterize different depression trajectories or subtypes, as well as their respective associations with functional outcomes, to inform future treatments. A more fine-grained approach, such as the experience sampling method (Myin-Germeys et al., 2018), may also be adopted to elucidate how personal-level and contextual factors can interact to maintain depressive symptoms and detect early warning signs of persistent and recurrent MDE. Incomplete data in a subsample of participants might have affected the observations, although findings from our imputed sample yielded generally similar results. We also acknowledge that the measure of dependent and independent SLEs is relatively generic in this study. Future work may consider adopting methods such as those described in Kender and Gardner (2010), wherein each participant is asked to rate the degree to which they consider an event related to their symptoms. With the experience of bullying being a persistent risk factor for nonsuicidal self-injury and mental health problems (Serafini et al., 2023), it may also be worthwhile to examine whether being a victim of bullying and a perpetrator would also contribute to the endurance of MDE in young people.

Lastly, the extensive societal changes that occurred during the time of data collection could have affected the phenomenology of MDE and its trajectory. The broadly consistent observations between our work and previous studies, nevertheless, suggest that core predictors of enduring MDE might be generally robust against the impact of large-scale external stressors. Replicating this study in other populations would be helpful to determine the generalizability of the present findings to other cultures and contexts.

## Conclusion

Around one-fifth of the general youth population in Hong Kong reported enduring MDE. On one hand, this rate appears lower than those observed in clinical populations and highlights the possibility of natural improvement of depressive symptomatology over time. On the other hand, the considerable individual and societal burdens that are associated with enduring MDE call for more targeted initiatives to improve the long-term outcomes of young people with early signs of a depressive disorder. Particularly in those with prior MDEs, more intensive care may be given to those presenting with comorbid symptoms of PTSD, anxiety, psychotic-like experiences, and eating disorders. Facilitating young people in developing more adaptive behaviors and cognitions when facing personally relevant life stressors would also be helpful. After all, only about one-third of those with an enduring course of MDE received any psychiatric or psychological help. Concerted efforts in early detection and intervention in ways that are acceptable to young people are urgently needed.

## Supporting information

Wong et al. supplementary material Wong et al. supplementary material

## Data Availability

De-identified participant data in anonymized form will be available upon reasonable request and should be directed to the corresponding authors (Stephanie MY Wong: stephanie@myinwong.com or Eric YH Chen: eychen.hk@gmail.com).

## Supplementary material

The supplementary material for this article can be found at http://doi.org/10.1017/S0033291725102468.
