# Supplementary material for: Determinants of enduring major depressive episodes in the youth population of Hong Kong: The roles of comorbid psychopathology and stressful life events
Source: Psychol Med. 2025 Nov 20;55:e352. doi: 10.1017/S0033291725102468 (PMC13058650; doi:10.1017/S0033291725102468)
Supplement: Wong et al. supplementary material [file S0033291725102468sup001.pdf]

Supplementary Material

Determinants of enduring major depressive episodes in the youth population of Hong Kong: the roles of comorbid psychopathology and stressful life events

Table S1. Details of the instruments used 2
Table S2. Missingness of variables 5
Table S3. Comparison of sociodemographic characteristics and rates of enduring MDE in participants with and without complete data 6
Table S4. Characteristics of the youth cohort with and without MDE at baseline in the complete-case and imputed samples 7
Table S5. Univariate and multivariable logistic regression exploring factors associated with enduring MDE in the complete-case sample 8
Table S6. Comparison of functioning and health-related quality of life at follow-up between remitted and enduring MDE 9
References 10

Table S1. Details of the instruments used

Variable	Time point	Measure used	Additional information
**Sociodemographics**			
Sex	Baseline	Questionnaire item	–
Age	Baseline	Questionnaire item	–
Any government subsidy received	Baseline	Questionnaire item	–
Any family psychiatric history	Baseline	Questionnaire item	An item asking participants whether their mother or father has ever been diagnosed with a psychiatric condition.
**Personality and psychological factors**			
Neuroticism	Baseline	Neuroticism subscale of the Big Five Inventory (BFI) [2,3]	Neuroticism is one of the most commonly studied personality traits for its role in mental health problems. The BFI–Neuroticism subscale comprises eight items as part of the 44-item BFI. Items were rated on a 5-point Likert scale from “disagree strongly” to “agree strongly”, wherein a higher score reflects higher levels of neuroticism.
Loneliness	Baseline	UCLA Loneliness Scale (Version 3) (UCLA-LS) [4,5]	20 items assessing perceived loneliness. Items were rated on a 4-point Likert scale from “never” to “often, wherein a higher score reflects higher loneliness.
Hopelessness	Baseline	Beck Hopelessness Scale (BHS) [6,7]	20 items assessing the level of hopelessness during the past week. Items were rated on a binary (true/false) checklist and summed to generate a composite score.
Impulsivity	Baseline	Barratt Impulsiveness Scale-11 (BIS-11) [8,9]	30 items assessing multiple dimensions of trait impulsivity, including attentional, motor, and non-planning impulsiveness. Items were rated on a 4-point Likert scale from “rarely/never” to “almost always/always”, wherein a higher score reflects higher impulsivity.
Resilience	Baseline	Connor-Davidson Resilience Scale 10-item (CD-RISC-10) [10,11]	10 items assessing perceived resilience even in the face of adversity. Items were rated on a 5-point Likert scale from “not true at all” to “true nearly all the time”, wherein a higher score reflects higher resilience.
**Clinical factors**			
Age at MDE onset	Baseline	CIDI–SC[12]	Participants identified to have lifetime MDE (through the CIDI-SC) were asked at around what age they first experienced such major depressive symptoms (e.g., depressive mood, lack of interest/pleasure in things) that lasted at least 2 weeks.
Depressive symptoms	Baseline	Patient Health Questionnaire (PHQ-9) [13]	Nine items corresponding to the DSM criteria for major depression, with each item rated on a 4-point Likert scale from “not at all” to “nearly every day”. A score of ≥10 reflects probable depression or a high depressive symptom level.
Anxiety symptoms	Baseline	Generalized Anxiety Disorder scale (GAD-7) [14]	Seven items corresponding to the DSM criteria for generalised anxiety disorder, with each item rated on a 4-point Likert scale from “not at all” to “nearly every day”. A score of ≥10 reflects probable anxiety disorder or high anxiety symptom levels.
Psychotic-like experiences	Baseline	Community Assessment of Psychic Experiences-Positive Scale (CAPE-P15) [15,16]	15 items capturing the frequency of positive psychotic-like experiences (PLEs) (covering persecutory ideation, bizarre experiences, and perceptual anomalies), as well as their associated distress, respectively (30 items total). On the two subscales, items were rated on 4-point Likert scales from “never” to “nearly always”, as well as “not distressed” to “very distressed”, respectively. Scores of ≥1.57 for frequency and ≥1.17 for distress reflect the presence of PLEs and elevated risk of psychotic symptoms.
PTSD symptoms	Baseline	Trauma Screening Questionnaire (TSQ) [17]	10 items capturing core symptoms of PTSD. A score of ≥6 reflects probable PTSD or high PTSD symptom levels.[17,18]
Alcohol dependence symptoms	Baseline	Alcohol Use Disorders Identification Test (AUDIT) [19]	10 items capturing risky or hazardous alcohol consumption, dependence symptoms, and probable alcohol use disorder. A score of ≥8 reflects high-risk alcohol dependence symptoms.[19]
Eating disorder symptoms	Baseline	Eating Disorder Examination-Questionnaire 6.0 (EDE-Q 6.0)[20]	22 items (after excluding the six items of frequency of eating disorder-related behaviours) capturing attitudes and feelings towards eating and body image during the past 28 days, with items rated on a 7-point scale (e.g., from “no days” to every day” and “not at all” to “markedly”). Four subscales (restraint, and concerns over weight, shape, and eating) can be derived from the items, which utilise mean scores rather than summed scores. The global EDE-Q score is derived from the sum of the four subscales, divided by four. A score of ≥2.3 reflects a probable eating disorder.[21]
Social anxiety symptoms	Baseline	Liebowitz Social Anxiety Scale (LSAS)[22]	24 items capturing a range of social interaction and performance scenarios that people might experience fear/anxiety, as well as avoid, respectively (48 items total). On the two subscales, items were rated on a 4-point scale from "none" to "severe", as well as "never (0%)" to "usually (67–100%)", respectively. A score of ≥60 reflects probable generalised social anxiety disorder.[23]
**Family and lifestyle factors**			

Poor family functioning	Baseline	Brief Family Relationship Scale (BFRS) [24]	16 items capturing family functioning, with each rated on a 4-point Likert scale from "completely agree" to "completely disagree", wherein a higher score reflects poorer family functioning.
Poor sleep quality	Baseline	Pittsburgh Sleep Quality Index (PSQI) [25,26]	19 items converted into seven major components, including subjective sleep quality, sleep latency, duration, efficiency, disturbances, use of sleep medication, and daytime dysfunction; a global index is generated, wherein a higher score reflects poorer sleep quality.
Frequent nightmares	Baseline	An item from the PSQI [27,28]	An item from the PSQI, "having bad dreams" (translates to "nightmares" in Chinese). Four options were given to capture the frequency of nightmares during the past month: (i) no nightmare, (ii) <1 time a week, (iii) 1–2 times a week, and (iv) ≥3 times a week. ≥1 nightmares a week reflects frequent nightmares, which is equivalent to moderate-to-severe levels of nightmare experiences in the DSM-V.
Days of moderate-to-vigorous intensity physical activity	Baseline	Two items from the International Physical Activity Questionnaire [1,29]	Number of days engaged in moderate intensity (e.g., carrying light loads, bicycling at a regular pace; excludes walking) or vigorous intensity (e.g., heavy lifting, aerobics, fast bicycling) physical activity during the past week. The sum of the two items reflects the number of days of moderate-to-vigorous intensity physical activity, with seven being the maximum number of days possible.
Problematic smartphone use	Baseline	Revised Chen Internet Addiction Scale (CIAS-R) [30–32]	26 items adapted from the CIAS-R to require the use of the Internet to be performed via smartphones for capturing problematic smartphone use (or smartphone overuse). The CIAS-R captures five key domains of behavioural addictions, including compulsive use, tolerance, withdrawal, problems with interpersonal relationships, and time management. Items were rated on a 4-point Likert scale from "not at all" to "very much". A score of ≥67 reflects problematic smartphone use.
**Distal and recent stressors**			
Any childhood adversity	Baseline	Items relevant to adverse childhood experiences adapted from the Composite International Diagnostic Interview (CIDI) 3.0 [33,34]	Captures four types of childhood adversity experienced before the age of 17 years, including emotional abuse (three items), physical abuse (two items), neglect (two items), and sexual abuse (two items); responses to each were rated on a 5-point scale from "never" to "very often". Any experience of childhood adversity was defined as a rating of 2 or above ("sometimes" to "very often") for any item.
Social unrest-related traumatic events	Baseline	Questionnaire items [1]	A checklist of three major experiences of traumatic events (TEs) since the emergence of the series of social unrest in Hong Kong (June 2019), including "crowd dispersal by the use of force", "arrest or detention", and "media viewing of others being physically attacked".
COVID-19 pandemic-related events	Baseline	Questionnaire items [1]	A checklist of four major experiences related to the COVID-19 pandemic since its outbreak in Hong Kong (January 2020), including "having sufficient gears" (reversed), "increased personal and rest time due to remote work/school" (reversed), "increased conflicts with family due to remote work/school", and "increased work/studies hours due to remote work/school". A 5-point Likert scale ("completely disagree" to "completely agree") was used before November 2020, which was replaced by a binary checklist thereafter. A rating of "completely disagree" or "disagree" for the items "having sufficient gears" and "increased personal and rest time" and a rating of "agree" or "completely agree" for the other two were recoded into 'yes' for those with their assessments completed before November 2020. A score of 0 was given to those who completed their assessments before the local outbreak.
Personal stressful life events	Follow-up	Adapted version of the List of Threatening Events (LTE) [35,36]	A binary (yes/no) checklist assessing exposure to 12 major personal life events during the past 12 months, with alternative options of "expelled from school' and "dropped out of school" for "sacked from job" and "unemployment", respectively, for the youth population, alongside with an "others" option. Two or more SLEs reflect high stress exposure.[1] Dependent SLEs included the following: "separation due to marital difficulties"; "serious problem with a close friend, neighbour or relative"; "unemployed/dropped out of school"; "sacked from job/expelled from school"; "major financial crisis"; "problems with police and court appearance"; and "broke off a steady relationship". Independent SLEs included the following: "serious illness, injury or assault to self"; "serious illness, injury or assault to a close relative"; "death of first-degree relative, including child or spouse"; "death of close family friend or second-degree relative"; "something valuable lost or stolen".
**Past-year service utilisation**			
Any past-year psychiatric/ psychological service use	Follow-up	Checklist of service providers [1]	Any mental health-related services obtained from psychiatrists, psychologists, or community psychiatric nurses during the past year.
**Functioning and HR-QoL**			
Days of reduced	Follow-up	Questionnaire items [1]	Two items capturing the number of days, during the past 30 days, the participant

and lost productivity			has experienced reduced and lost productivity at work/school due to psychiatric symptoms.
Social and occupational functioning	Follow-up	Social and Occupational Functioning Assessment Scale (SOFAS) [37]	Interviewer-rated instrument on impairments in social and occupational functioning due to mental health symptoms during the past six months. Ranges from 0 to 100, with a higher score reflecting more optimal functioning.
Health-related quality of life	Follow-up	12-Item Short Form Survey (SF-12) [38,39]	Comprises two component scores reflecting mental and physical health-related QoL (MCS-12, PCS-12).

Note. AUDIT=Alcohol Use Disorders Identification Test; BFI=Big Five Inventory; BFRS=Brief Family Relationship Scale; BHS=Beck Hopelessness Scale; BIS-11=Barratt Impulsiveness Scale-11; CAPE-P15=15-item Community Assessment of Psychic Experiences-Positive Scale; CD-RISC-10=Connor-Davidson Resilience Scale 10-item; CIAS-R=Revised Chen Internet Addiction Scale; CIDI-SC=Composite International Diagnostic Interview–Screening Scales; EDE-Q=Eating Disorder Examination Questionnaire; GAD-7=Generalized Anxiety Disorder scale; IPAQ=International Physical Activity Questionnaire; LSAS=Liebowitz Social Anxiety Scale; MDE=major depressive episode; PEs = COVID-19 pandemic-related events; PHQ-9=Patient Health Questionnaire; PSQI=Pittsburgh Sleep Quality Index; SLEs=personal stressful life events; TEs = social unrest-related traumatic events; TSQ=Trauma Screening Questionnaire; UCLA-LS=UCLA Loneliness Scale (Version 3).

Table S2. Missingness of variables

	% with incomplete data out of 1833 participants
**Enduring MDE**	
MDE at follow-up (CIDI-SC)	1.8%
**Sociodemographics**	
Female sex	0%
Age	0%
Any government subsidy received	0.2%
Any family psychiatric history	0.2%
**Personality and psychological factors**	
Neuroticism (BFI–Neuroticism)	0.4%
Loneliness (UCLA-LS)	2.8%
Hopelessness (BHS)	1.1%
Impulsivity (BIS-11)	2.7%
Resilience (CD-RISC-10)	0.5%
**Clinical factors**	
Age at first MDE onset	0%
Depressive symptoms (PHQ-9)	1%
Anxiety symptoms (GAD-7)	0.9%
Psychotic-like experiences (CAPE-P15)	0.3%
PTSD symptoms (TSQ)	0.2%
Alcohol dependence symptoms (AUDIT)	0%
Eating disorder symptoms (EDE-Q)	2.5%
Social anxiety symptoms (LSAS)	2.4%
**Family and lifestyle factors**	
Poor family functioning (BFRS)	0.1%
Poor sleep quality (PSQI)	0%
Frequent nightmares	0%
Physical activity days (IPAQ)	0%
Problematic smartphone use (CIAS-R)	0.4%
**Distal and recent stressors**	
Past exposure to childhood adversity	0%
Social unrest-related traumatic events	0%
COVID-19 pandemic-related events	1.4%
Recent personal stressors at follow-up (LTE)	1.6%
**Functioning at follow-up**	
Days of reduced productivity	1.7%
Days of lost productivity	1.7%
Social and occupational functioning (SOFAS)	4.4%
**Health-related QoL at follow-up**	
Physical HR-QoL (PCS-12)	1.5%
Mental HR-QoL (MCS-12)	1.5%
**Service utilisation at follow-up**	2.7%

Note. AUDIT=Alcohol Use Disorders Identification Test; BFI=Big Five Inventory; BFRS=Brief Family Relationship Scale; BHS=Beck Hopelessness Scale; BIS-11=Barratt Impulsiveness Scale-11; CAPE-P15=15-item Community Assessment of Psychic Experiences-Positive Scale; CD-RISC-10=Connor-Davidson Resilience Scale 10-item; CIAS-R=Revised Chen Internet Addiction Scale; CIDI-SC=Composite International Diagnostic Interview–Screening Scales; EDE-Q=Eating Disorder Examination Questionnaire; GAD-7=Generalized Anxiety Disorder scale; IPAQ=International Physical Activity Questionnaire; LSAS=Liebowitz Social Anxiety Scale; MDE=major depressive episode; PEs = COVID-19 pandemic-related events; PHQ-9=Patient Health Questionnaire; PSQI=Pittsburgh Sleep Quality Index; SLEs=personal stressful life events; TEs = social unrest-related traumatic events; TSQ=Trauma Screening Questionnaire; UCLA-LS=UCLA Loneliness Scale (Version 3).

Table S3. Comparison of sociodemographic characteristics and rates of enduring MDE in participants with and without complete data

Variable		*N*	With complete data (n = 1620)	Without complete data (n = 213)	*p*
Sex	Male	767	677 (41.8%)	90 (42.3%)	0.897
	Female	1066	943 (58.2%)	123 (57.7%)	
Age		1833	19.8 (2.7)	19.6 (2.7)	0.241
Any government subsidy received	No	1637	1448 (89.4%)	189 (90%)	0.784
	Yes	193	172 (10.6%)	21 (10%)	
Family psychiatric history	No	1603	1425 (88%)	178 (84.8%)	0.185
	Yes	227	195 (12%)	32 (15.2%)	
Enduring MDE	No	203	177 (79.7%)	26 (74.3%)	0.462
	Yes	54	45 (20.3%)	9 (25.7%)	

Note. Data are presented as n (%) or mean (SD). Chi-square tests or Mann-Whitney U tests were applied for the analyses. MDE=major depressive episode.

Table S4. Characteristics of the youth cohort with and without MDE at baseline in the complete-case and imputed samples

	Complete-case sample				Imputed sample			
	Whole sample (n = 1620)	Without MDE at baseline (n = 1398)	With MDE at baseline (n = 222)	*p*	Whole sample (n = 1833)	Without MDE at baseline (n = 1571)	With MDE at baseline (n = 262)	*p*
**Sociodemographics**								
Female sex	943 (58.2%)	**788 (56.4%)**	**155 (69.8%)**	**<.001**	**58.2%**	**56.3%**	**69.5%**	**<.001**
Age, mean (SD)	19.8 (2.7)	**19.8 (2.8)**	**20.2 (2.7)**	**0.022**	**19.8 (2.7)**	**19.74 (2.74)**	**20.15 (2.7)**	**0.012**
Any government subsidy received	172 (10.6%)	153 (10.9%)	19 (8.6%)	0.340	10.6%	10.6%	10%	0.985
Any family psychiatric history	195 (12%)	**146 (10.4%)**	**49 (22.1%)**	**<.001**	**12.4%**	**10.8%**	**22.3%**	**<.001**
**Personality and psychological factors**								
Neuroticism (BFI–Neuroticism), mean (SD)	25.47 (5.76)	**24.68 (5.51)**	**30.45 (4.71)**	**<.001**	**25.53 (5.80)**	**24.68 (5.54)**	**30.63 (4.58)**	**<.001**
Loneliness (UCLA-LS), mean (SD)	43.87 (8.94)	**43.01 (8.58)**	**49.31 (9.27)**	**<.001**	**44.12 (9.38)**	**43.04 (9)**	**49.45 (9.7)**	**<.001**
Hopelessness (BHS), mean (SD)	6.62 (4.04)	**6.19 (3.83)**	**9.32 (4.31)**	**<.001**	**6.67 (4.06)**	**6.22 (3.84)**	**9.4 (4.31)**	**<.001**
Impulsivity (BIS-11), mean (SD)	63.13 (8.41)	**62.69 (8.31)**	**65.91 (8.55)**	**<.001**	**63.34 (8.82)**	**62.73 (8.61)**	**65.65 (9.13)**	**<.001**
Resilience (CD-RISC-10), mean (SD)	24.21 (6.34)	**24.86 (6.02)**	**20.11 (6.78)**	**<.001**	**24.14 (6.36)**	**24.85 (6.04)**	**19.9 (6.57)**	**<.001**
**Clinical factors**								
Age at first MDE onset (CIDI E3), mean (SD)	–	–	15.9 (4.1)	–	–	–	15.94 (3.97)	–
Depressive symptoms (PHQ-9 ≥ 10)	382 (23.6%)	**249 (17.8%)**	**133 (59.9%)**	**<.001**	**23.9%**	**18.1%**	**58.9%**	**<.001**
Anxiety symptoms (GAD-7 ≥ 10)	210 (13%)	**121 (8.7%)**	**89 (40.1%)**	**<.001**	**13.1%**	**9%**	**37.7%**	**<.001**
PLEs (CAPE-P15 frequency ≥1.57 and distress ≥ 1.17)	139 (8.6%)	**94 (6.7%)**	**45 (20.3%)**	**<.001**	**8.9%**	**7%**	**20%**	**<.001**
PTSD symptoms (TSQ ≥ 6)	50 (3.1%)	**32 (2.3%)**	**18 (8.1%)**	**<.001**	**3.%**	**2.2%**	**7.6%**	**<.001**
Alcohol dependence symptoms (AUDIT ≥ 8)	94 (5.8%)	**73 (5.2%)**	**21 (9.5%)**	**0.019**	5.7%	5.3%	8.4%	0.176
Eating disorder symptoms (EDE-Q mean ≥ 2.3)	207 (12.8%)	**165 (11.8%)**	**42 (18.9%)**	**0.004**	**13.2%**	**12.4%**	**18.2%**	**0.049**
Social anxiety symptoms (LSAS ≥ 60)	268 (16.5%)	**195 (13.9%)**	**73 (32.9%)**	**<.001**	**16.5%**	**13.9%**	**32.2%**	**<.001**
**Family and lifestyle factors**								
Poor family functioning (BFRS), mean (SD)	19.68 (7.04)	**19.19 (6.71)**	**22.77 (8.23)**	**<.001**	**19.73 (7.05)**	**19.18 (6.69)**	**22.98 (8.22)**	**<.001**
Poor sleep quality (PSQI), mean (SD)	5.49 (3.21)	**5.15 (3)**	**7.64 (3.65)**	**<.001**	**5.60 (3.28)**	**5.23 (3.05)**	**7.86 (3.69)**	**<.001**
Frequent nightmares (≥1/week)	258 (15.5%)	**167 (11.9%)**	**76 (34.2%)**	**<.001**	**16.1%**	**13%**	**34.4%**	**<.001**
Physical activity days (IPAQ), mean (SD)	3.13 (2.47)	**3.18 (2.48)**	**2.77 (2.36)**	**0.026**	**3.14 (2.46)**	**3.21 (2.47)**	**2.69 (2.36)**	**0.001**
Problematic smartphone use (CIAS-R ≥ 67)	494 (29.8%)	**377 (27%)**	**107 (48.2%)**	**<.001**	**30%**	**26.9%**	**48.6%**	**<.001**
**External stressors**								
Any past exposure to childhood adversity	568 (35.1%)	**443 (31.7%)**	**125 (56.3%)**	**<.001**	**36.1%**	**32.5%**	**57.6%**	**<.001**
≥2 social unrest-related TEs at baseline	313 (19.3%)	**257 (18.4%)**	**56 (25.2%)**	**0.021**	18.4%	17.6%	23.7%	0.076
≥2 COVID-19 PEs at baseline	547 (35.6%)	**484 (36.3%)**	**90 (44.3%)**	**0.034**	36.9%	35.8%	42.9%	0.150

Note. Data are presented as n (%) or mean (SD). AUDIT=Alcohol Use Disorders Identification Test; BFI=Big Five Inventory; BFRS=Brief Family Relationship Scale; BHS=Beck Hopelessness Scale; BIS-11=Barratt Impulsiveness Scale-11; CAPE-P15=15-item Community Assessment of Psychic Experiences-Positive Scale; CD-RISC-10=Connor-Davidson Resilience Scale 10-item; CIAS-R=Revised Chen Internet Addiction Scale; EDE-Q=Eating Disorder Examination Questionnaire; GAD-7=Generalized Anxiety Disorder scale; IPAQ=International Physical Activity Questionnaire; LSAS=Liebowitz Social Anxiety Scale; MDE=major depressive episode; PHQ-9=Patient Health Questionnaire; PSQI=Pittsburgh Sleep Quality Index; SLEs=personal stressful life events; TSQ=Trauma Screening Questionnaire; UCLA-LS=UCLA Loneliness Scale (Version 3).

† Absence of MDE at 1-year follow-up, assessed using the CIDI-SC, among those with 12-month MDE at baseline.

* Presence of MDE at 1-year follow-up, among those with 12-month MDE at baseline.

Missing from 85 participants.

Table S5. Univariate and multivariable logistic regression exploring factors associated with enduring MDE in the complete-case sample

	Enduring MDE at follow-up			
	Unadjusted OR (95% CI)	*p*	Adjusted OR† (95% CI)	*p*
**Sociodemographics**				
Female sex	1.08 (0.53-2.22)	0.833	1.05 (0.51-2.17)	0.891
Age	1.16 (0.83-1.63)	0.378	1.20 (0.84-1.69)	0.314
Any government subsidy received	1.46 (0.5-4.28)	0.495	1.62 (0.54-4.90)	0.389
Any family psychiatric history	1.01 (0.46-2.22)	0.978	1.00 (0.46-2.21)	0.992
**Personality and psychological factors**				
Neuroticism (BFI–Neuroticism)	**1.59 (1.03-2.46)**	**0.036**	**1.59 (1.03-2.46)**	**0.038**
Loneliness (UCLA-LS)	1.33 (0.97-1.83)	0.079	1.34 (0.97-1.86)	0.075
Hopelessness (BHS)	**1.40 (1.02-1.92)**	**0.036**	**1.41 (1.02-1.94)**	**0.035**
Impulsivity (BIS-11)	1.14 (0.83-1.57)	0.431	1.17 (0.84-1.63)	0.347
Resilience (CD-RISC-10)	0.78 (0.57-1.06)	0.116	0.78 (0.57-1.07)	0.120
**Clinical factors**				
Age at first MDE onset (CIDI E3)	0.92 (0.69-1.23)	0.566	0.85 (0.62-1.16)	0.302
Depressive symptoms (PHQ-9 ≥ 10)	**4.69 (1.99-11.06)**	**<.001**	**4.89 (2.05-11.68)**	**<.001**
Anxiety symptoms (GAD-7 ≥ 10)	**2.48 (1.27-4.82)**	**0.008**	**2.52 (1.28-4.97)**	**0.007**
PLEs (CAPE-P15 freq. ≥1.57 and distress ≥ 1.17)	1.84 (0.87-3.9)	0.111	2.00 (0.93-4.34)	0.078
PTSD symptoms (TSQ ≥ 6)	**4.67 (1.73-12.58)**	**0.002**	**4.63 (1.70-12.62)**	**0.003**
Alcohol dependence symptoms (AUDIT ≥ 8)	1.66 (0.61-4.56)	0.324	1.67 (0.59-4.68)	0.331
Eating disorder symptoms (EDE-Q mean ≥ 2.3)	**2.4 (1.14-5.08)**	**0.022**	**2.46 (1.14-5.32)**	**0.022**
Social anxiety symptoms (LSAS ≥ 60)	1.16 (0.58-2.31)	0.669	1.12 (0.56-2.25)	0.746
**Family and lifestyle factors**				
Poor family functioning (BFRS)	1.25 (0.94-1.66)	0.122	1.22 (0.92-1.63)	0.165
Poor sleep quality (PSQI)	0.93 (0.69-1.25)	0.628	0.92 (0.68-1.24)	0.581
Frequent nightmares (≥1/week)	1.54 (0.79-3.01)	0.208	1.59 (0.80-3.15)	0.183
Physical activity days (IPAQ)	0.82 (0.57-1.17)	0.264	0.83 (0.58-1.20)	0.318
Problematic smartphone use (CIAS-R ≥ 67)	0.83 (0.43-1.6)	0.573	0.82 (0.42-1.61)	0.568
**Distal and recent stressors**				
Any past exposure to childhood adversity	1.21 (0.62-2.35)	0.576	1.15 (0.57-2.32)	0.689
≥2 social unrest-related TEs at baseline				
≥2 COVID-19 PEs at baseline				
Recent stress exposure at follow-up (≥2 SLEs)	**2.77 (1.38-5.53)**	**0.004**	**2.94 (1.45-5.99)**	**0.003**
≥2 independent SLEs at follow-up	1.28 (0.48-3.42)	0.623	1.36 (0.50-3.69)	0.550
≥2 dependent SLEs at follow-up	**4.45 (1.81-10.91)**	**0.001**	**4.46 (1.80-11.05)**	**0.001**

Note. Data are presented as n (%) or mean (SD). AUDIT=Alcohol Use Disorders Identification Test; BFI=Big Five Inventory; BFRS=Brief Family Relationship Scale; BHS=Beck Hopelessness Scale; BIS-11=Barratt Impulsiveness Scale-11; CAPE-P15=15-item Community Assessment of Psychic Experiences-Positive Scale; CD-RISC-10=Connor-Davidson Resilience Scale 10-item; CIAS-R=Revised Chen Internet Addiction Scale; EDE-Q=Eating Disorder Examination Questionnaire; GAD-7=Generalized Anxiety Disorder scale; IPAQ=International Physical Activity Questionnaire; LSAS=Liebowitz Social Anxiety Scale; MDE=major depressive episode; PHQ-9=Patient Health Questionnaire; PSQI=Pittsburgh Sleep Quality Index; SLEs=personal stressful life events; TSQ=Trauma Screening Questionnaire; UCLA-LS=UCLA Loneliness Scale (Version 3).
† Adjusted for sex, age, socioeconomic status, and family psychiatric history.

Table S6. Comparison of functioning and health-related quality of life at follow-up between remitted and enduring MDE

	Whole sample (n = 1833)	Remitted MDE (78.7%)	Enduring MDE (21.3%)	Statistics
Functioning				
Days of reduced productivity	2.46 (4.96)	**4.10 (6.10)**	**9.28 (9.75)**	***Z* = -4.15, *p* < .001**
Days of lost productivity	0.44 (2.38)	**0.65 (2.56)**	**3.75 (7.22)**	***Z* = -3.49, *p* < .001**
Social and occupational functioning (SOFAS)	77.32 (9.39)	**75.39 (9.31)**	**65.76 (11.28)**	***Z* = -5.65, *p* < .001**
Health-related QoL				
Physical HR-QoL (PCS-12)	53.26 (6.02)	51.25 (7.12)	49.85 (8.52)	*Z* = -0.95, *p* = 0.171
Mental HR-QoL (MCS-12)	47.45 (9.53)	**43.08 (9.72)**	**34.00 (13.61)**	***Z* = -4.47, *p* < .001**

Note. Analyses were conducted on the imputed sample (n = 1833). MCS-12=mental component of the 12-Item Short Form Health Survey (SF-12); PCS-12=physical component of SF-12; HR-QoL=health-related quality of life; SOFAS=Social and Occupational Functioning Assessment Scale.

References

1. Wong SMY, Chen EYH, Suen YN, Wong CSM, Chang WC, Chan SKW, et al. Prevalence, time trends, and correlates of major depressive episode and other psychiatric conditions among young people amid major social unrest and COVID-19 in Hong Kong: a representative epidemiological study from 2019 to 2022. Lancet Reg Health West Pac. 2023 Nov;40:100881.

2. John OP, Donahue EM, Kentle RL. Big five inventory [Internet]. PsycTESTS Dataset. American Psychological Association (APA); 2012. Available from: https://psycnet.apa.org/doiLanding?doi=10.1037/t07550-000

3. So MM, Suen YN, Wong SMY, Cheung C, Chan SKW, Lee EHM, et al. Resilient, undercontrolled, and overcontrolled personality types in Hong Kong youths and the association with mental health outcomes. J Pers. 2023 Sep 18;(00):1–14.

4. Russell DW. UCLA Loneliness Scale (Version 3): reliability, validity, and factor structure. J Pers Assess. 1996 Feb;66(1):20–40.

5. Cole A, Bond C, Qualter P, Maes M. A Systematic Review of the Development and Psychometric Properties of Loneliness Measures for Children and Adolescents. Int J Environ Res Public Health. 2021 Mar 22;18(6):3285.

6. Beck AT, Weissman A, Lester D, Trexler L. The measurement of pessimism: the hopelessness scale. J Consult Clin Psychol. 1974 Dec;42(6):861–5.

7. Liu XC, Chen H, Liu ZZ, Wang JY, Jia CX. Prevalence of suicidal behaviour and associated factors in a large sample of Chinese adolescents. Epidemiol Psychiatr Sci. 2019 Jun;28(3):280–9.

8. Patton JH, Stanford MS, Barratt ES. Factor structure of the Barratt impulsiveness scale. J Clin Psychol. 1995 Nov;51(6):768–74.

9. Lu CF, Jia CX, Xu AQ, Dai AY, Qin P. Psychometric characteristics of Chinese version of Barratt Impulsiveness Scale-11 in suicides and living controls of rural China. Omega. 2012;66(3):215–29.

10. Campbell-Sills L, Stein MB. Psychometric analysis and refinement of the Connor-davidson Resilience Scale (CD-RISC): Validation of a 10-item measure of resilience. J Trauma Stress. 2007 Dec;20(6):1019–28.

11. Rui S, Yang X, Lau MM, Lau JTF. Psychometric properties and normative data of the 10-item Connor–Davidson Resilience Scale among Chinese adolescent students in Hong Kong. Child Psychiatry and Human Development. 2020 Dec;51(6):925–33.

12. Kessler RC, Calabrese JR, Farley PA, Gruber MJ, Jewell MA, Katon W, et al. Composite International Diagnostic Interview screening scales for DSM-IV anxiety and mood disorders. Psychol Med. 2013 Aug;43(8):1625–37.

13. Kroenke K, Spitzer RL, Williams JBW. The PHQ-9. J Gen Intern Med. 2001 Sep;16(9):606–13.

14. Spitzer RL, Kroenke K, Williams JBW, Löwe B. A brief measure for assessing generalized anxiety disorder: the GAD-7. Arch Intern Med. 2006 May 22;166(10):1092–7.

15. Capra C, Kavanagh DJ, Hides L, Scott J. Brief screening for psychosis-like experiences. Schizophr Res. 2013 Sep;149(1-3):104–7.

16. Sun M, Wang D, Jing L, Yang N, Zhu R, Wang J, et al. Comparisons between self-reported and interview-verified psychotic-like experiences in adolescents. Early Interv Psychiatry. 2022 Jan;16(1):69–77.

17. Brewin CR, Rose S, Andrews B, Green J, Tata P, McEvedy C, et al. Brief screening instrument for post-traumatic stress disorder. Br J Psychiatry. 2002 Aug;181:158–62.

18. Wong SMY, Hui CLM, Wong CSM, Suen YN, Chan SKW, Lee EHM, et al. Prospective prediction of PTSD and depressive symptoms during social unrest and COVID-19 using a brief online tool. Psychiatry Res. 2021 Apr;298:113773.

19. Saunders JB, Aasland OG, Babor TF, de la Fuente JR, Grant M. Development of the Alcohol Use Disorders Identification Test (AUDIT): WHO Collaborative Project on Early Detection of Persons with Harmful Alcohol Consumption--II. Addiction. 1993 Jun;88(6):791–804.

20. Fairburn CG. Cognitive Behavior Therapy and Eating Disorders. Guilford Press; 2008. 324 p.

21. Mond JM, Hay PJ, Rodgers B, Owen C, Beumont PJV. Validity of the Eating Disorder Examination Questionnaire (EDE-Q) in screening for eating disorders in community samples. Behav Res Ther. 2004 May;42(5):551–67.

22. Heimberg RG, Horner KJ, Juster HR, Safren SA, Brown EJ, Schneier FR, et al. Psychometric properties of the Liebowitz Social Anxiety Scale. Psychol Med. 1999 Jan;29(1):199–212.

23. Mennin DS, Fresco DM, Heimberg RG, Schneier FR, Davies SO, Liebowitz MR. Screening for social anxiety disorder in the clinical setting: using the Liebowitz Social Anxiety Scale. J Anxiety Disord. 2002;16(6):661–73.

24. Fok CCT, Allen J, Henry D, People Awakening Team. The brief family relationship scale: a brief measure of the relationship dimension in family functioning. Assessment. 2014 Feb;21(1):67–72.

25. Guo S, Sun W, Liu C, Wu S. Structural Validity of the Pittsburgh Sleep Quality Index in Chinese Undergraduate Students. Front Psychol. 2016 Aug 8;7:1126.

26. Buysse DJ, Reynolds CF 3rd, Monk TH, Berman SR, Kupfer DJ. The Pittsburgh Sleep Quality Index: a new instrument for psychiatric practice and research. Psychiatry Res. 1989 May;28(2):193–213.

27. Wong SMY, Hui CLM, Cheung VKW, Suen YN, Chan SKW, Lee EHM, et al. Prevalence of frequent nightmares and their prospective associations with 1-year psychiatric symptoms and disorders and functioning in young adults: a large-scale epidemiological study in Hong Kong. Sleep. 2023 Apr 12;46(4):zsac296.

28. Li SX, Zhang B, Li AM, Wing YK. Prevalence and correlates of frequent nightmares: a community-based 2-phase study. Sleep. 2010 Jun;33(6):774–80.

29. Macfarlane D, Chan A, Cerin E. Examining the validity and reliability of the Chinese version of the International Physical Activity Questionnaire, long form (IPAQ-LC). Public Health Nutr. 2011 Mar;14(3):443–50.

30. Mak KK, Lai CM, Ko CH, Chou C, Kim DI, Watanabe H, et al. Psychometric properties of the Revised Chen Internet Addiction Scale (CIAS-R) in Chinese adolescents. J Abnorm Child Psychol. 2014 Oct;42(7):1237–45.

31. Chen SH, Weng LJ, Su YJ, Wu HM, Yang PF. Development of Chinese Internet Addiction Scale and its psychometric study. Chinese Journal of Psychology. 2003 Jan 1;45(3):279–94.

32. Wong SM, Chen EY, Wong CS, Suen YN, Chan DL, Tsang SH, et al. Impact of smartphone overuse on 1-year severe depressive symptoms and momentary negative affect: Longitudinal and experience sampling findings from a representative epidemiological youth sample in Hong Kong. Psychiatry Res. 2022 Nov 2;318:114939.

33. Kessler RC, Ustün TB. The World Mental Health (WMH) Survey Initiative Version of the World Health Organization (WHO) Composite International Diagnostic Interview (CIDI). Int J Methods Psychiatr Res. 2004;13(2):93–121.

34. Mall S, Mortier P, Taljaard L, Roos J, Stein DJ, Lochner C. The relationship between childhood adversity, recent stressors, and depression in college students attending a South African university. BMC Psychiatry. 2018 Mar 9;18(1):63.

35. Brugha T, Bebbington P, Tennant C, Hurry J. The List of Threatening Experiences: a subset of 12 life event categories with considerable long-term contextual threat. Psychol Med. 1985 Feb;15(1):189–94.

36. Wong SMY, Ip CH, Hui CLM, Suen YN, Wong CSM, Chang WC, et al. Prevalence and correlates of suicidal behaviours in a representative epidemiological youth sample in Hong Kong: the significance of suicide-related rumination, family functioning, and ongoing population-level stressors. Psychol Med. 2022 Jun 2;1–11.

37. Goldman HH, Skodol AE, Lave TR. Revising axis V for DSM-IV: a review of measures of social functioning. Am J Psychiatry. 1992 Sep;149(9):1148–56.

38. Ware J Jr, Kosinski M, Keller SD. A 12-Item Short-Form Health Survey: construction of scales and preliminary tests of reliability and validity. Med Care. 1996 Mar;34(3):220–33.

39. Lam CLK, Tse EYY, Gandek B. Is the standard SF-12 health survey valid and equivalent for a Chinese population? Qual Life Res. 2005 Mar;14(2):539–47.
